# Diagnosis of Alzheimer’s Disease in Clinical Practice: Time to Incorporate Biomarkers?

**DOI:** 10.3233/JAD-240660

**Published:** 2024-10-08

**Authors:** Martin Vyhnalek, Martina Laczó, Jan Laczó

**Affiliations:** Memory Clinic, Department of Neurology, Second Faculty of Medicine, Charles University and Motol University Hospital, Prague, Czechia

**Keywords:** Alzheimer’s disease, behavioral variant frontotemporal dementia, cerebrospinal fluid, Lewy body dementia, limbic-predominant age-related TDP-43 encephalopathy, mild cognitive impairment, positron emission tomography, primary age-related tauopathy, subjective cognitive decline, suspected non-Alzheimer’s disease pathophysiology

## Abstract

Hippocampal dysfunction is associated with early clinical signs of Alzheimer’s disease (AD). Due to the limited availability or invasiveness of current biomarkers, the AD diagnosis is usually based on cognitive assessment and structural brain imaging. The recent study by Lalive and colleagues examined the specificity of brain morphometry for the AD diagnosis in a memory clinic cohort with hippocampal-type amnestic syndrome. The results indicate that memory deficits and hippocampal atrophy are similar in AD and non-AD patients, highlighting their low diagnostic specificity. These findings challenge the traditional AD diagnosis and underscore the need for biomarkers to differentiate specific neuropathological entities.

The recent study by Lalive and colleagues compared hippocampal volumes in biomarker-defined patients with and without Alzheimer’s disease (AD) who presented with amnestic syndrome of the hippocampal type (ASHT) and found that comparable memory deficits and hippocampal atrophy were present in both AD and non-AD patients, but the specificity for the AD diagnosis based on these criteria was low, thus highlighting the need for more reliable biomarkers.^1^ Early clinical signs of AD are closely linked to hippocampal dysfunction, and prior to the biomarker era, the AD diagnosis was based on the combination of cognitive impairment with a predominant amnestic syndrome and hippocampal atrophy.^2^ This approach is still used in clinical practice because blood-based biomarkers have not been validated for routine clinical use and the currently available AD biomarkers are difficult to access (e.g., amyloid positron emission tomography [PET] imaging) or invasive (e.g., cerebrospinal fluid [CSF] biomarkers).^3^ Memory assessment is an important tool for assessing progression to AD dementia in patients with mild cognitive impairment (MCI), subjective cognitive decline, and cognitively healthy older adults.^4,5^ Progression to AD dementia can be predicted with greater accuracy when age and cognitive measures are combined with hippocampal volume measurements. Memory deficits in MCI due to AD have been associated with hippocampal atrophy,^6^ which can be assessed clinically using visual rating scales.^7^ However, post-mortem studies have shown that the specificity of clinical AD diagnosis in expert centers ranges from 44.3% to 70.8%, highlighting the limitations of conventional diagnostic approaches.^8^

The low diagnostic accuracy of standard diagnostic procedures may be due to challenges in interpreting memory impairment and hippocampal atrophy. While memory impairment is sensitive to AD pathology, its specificity is limited because profound episodic memory impairment is present in other dementias, such as behavioral variant frontotemporal dementia (bvFTD) and Lewy body dementia (LBD).^9,10^ To increase the specificity of cognitive assessment for the AD diagnosis, the ASHT, defined as low free recall that does not improve with cueing despite the controlled encoding, has been postulated.^11^ Although this pattern predicts progression to AD dementia in MCI patients and correlates with hippocampal volume, neither cross-sectional nor longitudinal studies have confirmed the superiority of the tests using controlled encoding and cued recall over standard memory tests.^12,13^ A similar problem of low specificity applies to hippocampal atrophy, which is an important feature of other dementias such as bvFTD, LBD, or semantic variant of primary progressive aphasia.^14–16^ It is worth noting that neuropathological studies have shown that medial temporal lobe (MTL) atrophy is associated with dementia, but is not specific to individual pathologies.^17^

Emerging disease-modifying therapies for AD have increased the urgent need for its early and accurate diagnosis. Diagnosis is particularly challenging in neurodegenerative diseases where memory impairment and hippocampal atrophy are dominant features. Such a group of neurodegenerative diseases in which amyloid-β is absent (i.e., with negative amyloid PET imaging or normal CSF amyloid-β) is referred to as suspected non-Alzheimer’s disease pathophysiology (SNAP), and common examples include primary age-related tauopathy (PART) and limbic-predominant age-related TDP-43 encephalopathy (LATE).^18^

PART is characterized by neurofibrillary tangles that accumulate predominantly in the MTL in the absence of amyloid plaques.^19^ Clinically, PART presents with slowly progressive memory impairment, but slower psychomotor speed and executive dysfunction may be present. Importantly, PART typically presents as an MCI syndrome without progression to dementia because the distribution of pathological changes is limited to the MTL. PET imaging has been shown to be a useful diagnostic tool for PART, with a negative amyloid PET imaging indicating the absence of amyloid pathology, and a positive tau PET imaging indicating the presence of tau pathology confined to the MTL.

LATE is characterized by TDP-43 pathology predominantly in the MTL with or without coexisting hippocampal sclerosis.^20^ LATE is most common in older adults (≥75 years) and clinically presents with slowly progressive cognitive impairment, primarily in episodic memory and less so in semantic memory, with relatively spared cortical cognitive functions. Hippocampal atrophy is disproportionate to the severity of the syndrome and is typically more pronounced in the anterior hippocampus. A typical biomarker profile is characterized by the absence of amyloid and tau pathology, as indicated by negative amyloid PET imaging or normal CSF amyloid-β, and negative tau PET imaging or normal CSF phosphorylated tau, respectively. A promising biomarker is the typical profile of MTL hypometabolism on ^18^F-fluorodeoxyglucose-PET imaging, as opposed to hypometabolism in the inferior temporal gyrus and parietal cortex in AD.^21^ Although there is no specific biomarker for LATE, the combination of clinical, neuroimaging, and CSF findings allows the diagnosis to be made with some confidence.

The recent study by Lalive and colleagues in the memory clinic population adds to the current knowledge of the differential diagnosis of AD, SNAP, and other conditions leading to cognitive decline.^1^ The authors used a Free and Cued Selective Reminding Test (FCSRT),^1^ previously validated and recommended for the diagnosis of hippocampal dysfunction, and enrolled 92 biomarker-defined memory clinic patients with ASHT characterized by a previously validated cutoff below 40/48 on cued recall. More than half of the patients were diagnosed with MCI, while the remaining patients were diagnosed with dementia. The characteristics of the included participants highlight the low specificity of the proposed ASHT criterion for the AD diagnosis. Notably, only 35% of patients met the biomarker criteria for AD, as evidenced by CSF amyloid-β and phosphorylated tau positivity. The remaining 65% were amyloid-negative and therefore referred to as non-AD group. Within this group, 33% were classified as SNAP, with the remaining patients having various etiologies, including cerebrovascular disease, bvFTD, and LBD. There were no differences between the AD and non-AD groups in Mini-Mental State Examination score, FCSRT subscores, or any regional brain MRI measures, including the hippocampus. Smaller hippocampal volume was associated with memory impairment regardless of diagnosis. In this respect, the study builds on previous research showing pronounced MTL atrophy in non-AD dementias and suggesting a low specificity of hippocampal atrophy for the AD diagnosis.^14–16^ In conclusion, these findings challenge the traditional clinical AD diagnosis based on memory impairment with ASHT and hippocampal atrophy, and pave the way for a more accurate, biologically based diagnosis using biomarkers to differentiate specific neuropathological entities.

These results are consistent with the National Institute on Aging and the Alzheimer’s Association (NIA-AA) recommendations published in 2018 and the Alzheimer’s Association Workgroup revised criteria published in 2024,^22,23^ which represent significant advances in the biological definition of AD, as well as with the recent recommendations of the European multidisciplinary taskforce, which emphasize the importance of biomarkers in the diagnostic process of neurocognitive disorders.^24^ All of these recommendations support our argument for the integration of biomarkers to improve the diagnostic accuracy in AD. It should be noted that CSF biomarkers, amyloid and tau PET imaging, and blood-based biomarkers have distinct advantages over cognitive testing and brain imaging in the AD diagnosis. While cognitive testing and brain imaging provide valuable information about brain function and structure, biomarkers reflecting AD pathophysiology can provide specific insights into the underlying pathological processes, such as amyloid-β and tau deposition. This specificity makes these biomarkers critical for early and accurate diagnosis, especially in cases where traditional methods show overlap between different neurodegenerative diseases. Until recently developed blood-based biomarkers are fully validated, the AD diagnosis should include amyloid PET imaging and CSF biomarkers, which are currently the most reliable measures of AD pathology in clinical practice. This approach ensures that we are using the best tools available while keeping an eye on future developments in blood-based biomarkers. The integration of biomarkers is critical to improving diagnostic accuracy, but the role of the clinical expert, incorporating clinical information and the results of neurological, cognitive testing and brain imaging, is still indispensable. As recently highlighted, the advent of new diagnostic tools and treatments, such as plasma biomarkers and anti-amyloid therapies, will require continued reliance on the clinical acumen of physicians to navigate and apply these advances effectively.^22,25^

## AUTHOR CONTRIBUTIONS

Martin Vyhnalek (Writing – original draft; Writing – review & editing); Martina Laczó (Writing – original draft; Writing – review & editing); Jan Laczó (Writing – original draft; Writing – review & editing).
